# Hypothermic Machine Perfusion Reduces Delayed Graft Function and Improves One-Year Graft Survival of Kidneys from Expanded Criteria Donors: A Meta-Analysis

**DOI:** 10.1371/journal.pone.0081826

**Published:** 2013-12-10

**Authors:** Baoping Jiao, Shurong Liu, Hao Liu, Donghua Cheng, Ying Cheng, Yongfeng Liu

**Affiliations:** Department of General Surgery, The First Hospital of China Medical University, Shenyang, China; UNIFESP Federal University of São Paulo, Brazil

## Abstract

**Background:**

Expanded criteria donors (ECDs) are currently accepted as potential sources to increase the donor pool and to provide more chances of kidney transplantation for elderly recipients who would not survive long waiting periods. Hypothermic machine perfusion (HMP) is designed to mitigate the deleterious effects of simple cold storage (CS) on the quality of preserved organs, particularly when the donor is in a marginal status.

**Methods:**

We compared the transplant outcomes in patients receiving ECD kidneys with either HMP or CS graft preservation. Articles from the MEDLINE, EMBASE and Cochrane Library databases were searched and all studies reporting outcomes from HMP versus CS methods of kidney preservation were included in this meta-analysis. The parameters analyzed included the incidence of delayed graft function (DGF), primary non-function (PNF) and one-year graft and patient survival.

**Results:**

A total of seven studies qualified for the review, involving 2374 and 8716 kidney grafts with HMP or CS preservation respectively, all from ECD donors. The incidence of delayed graft function (DGF) was significantly reduced with an odd ratio(OR) of 0.59 (95% CI 0.54–0.66, P<0.001) and one-year graft survival was significantly improved with an OR of 1.12 (95% CI 1.03–1.21, P = 0.005) in HMP preservation compared to CS. However, there was no difference in the incidence of PNF (OR 0.54, 95% CI 0.21–1.40, P = 0.20), and one-year patient survival (OR 0.98, 95% CI 0.94–1.02, P = 0.36) between HMP and CS preservation.

**Conclusions:**

HMP was associated with a reduced incidence of DGF and an with increased one-year graft survival, but it was not associated with the incidence of PNF and one-year patient survival.

## Introduction

Kidney transplantation is the optimal treatment for patients with end-stage renal disease(ESRD) [1]. Because of a persistent donor organ shortage, kidneys from expanded criteria donors (ECDs) are currently accepted by many centers and have been successfully transplanted to increase the donor pool [2], [3], thereby facilitating timely kidney transplantation for elderly recipients who would not survive long waiting periods [4], [5] ECDs are defined as allografts from deceased donors older than 60 years of age and those from donors aged 50–59 years old with at least two of the followings characteristics: history of hypertension, serum creatinine greater than 1.5 mg/dL or cerebrovascular as the cause of death[6]. Compared with standard criteria donor(SCD) kidneys, kidneys from ECDs can be associated with a higher rate of delayed graft function(DGF), primary non-function(PNF),acute rejection and a more complicated postoperative course, resulting in inferior long-term graft survival overall [7]–[10]. Although ECD kidneys have an overall 1.7 times greater risk for graft failure [3], it has also been shown that transplantation of these kidneys has a significant survival benefit when compared with dialysis treatment[11], especially for elderly recipients [12].

To maximize the benefit of donated kidneys, two kidney preservation methods have been developed over the past 30 years, namely hypothermic machine perfusion (HMP) and static cold storage (CS)[13]. Static cold storage, with solutions designed in the 1980s, remains the gold standard in kidney transplantation. However, it has been reported that SC was unable to fully protect ECD kidneys, while HMP could mitigate the deleterious effects of CS, reducing the incidence of DGF for ECD kidney transplantations [14], [15].

To better understand whether HMP could obtain better outcomes in ECD kidney transplantation compared to CS, We conducted a systematic review and meta-analysis of the available studies. We assessed the impact of HMP on rates of DGF, PNF and one-year graft and patient survival. These data could help clinical transplant professionals to decide the best way to preserve ECD kidneys.

## Materials and Methods

### Data sources and searches

**Figure 1:**
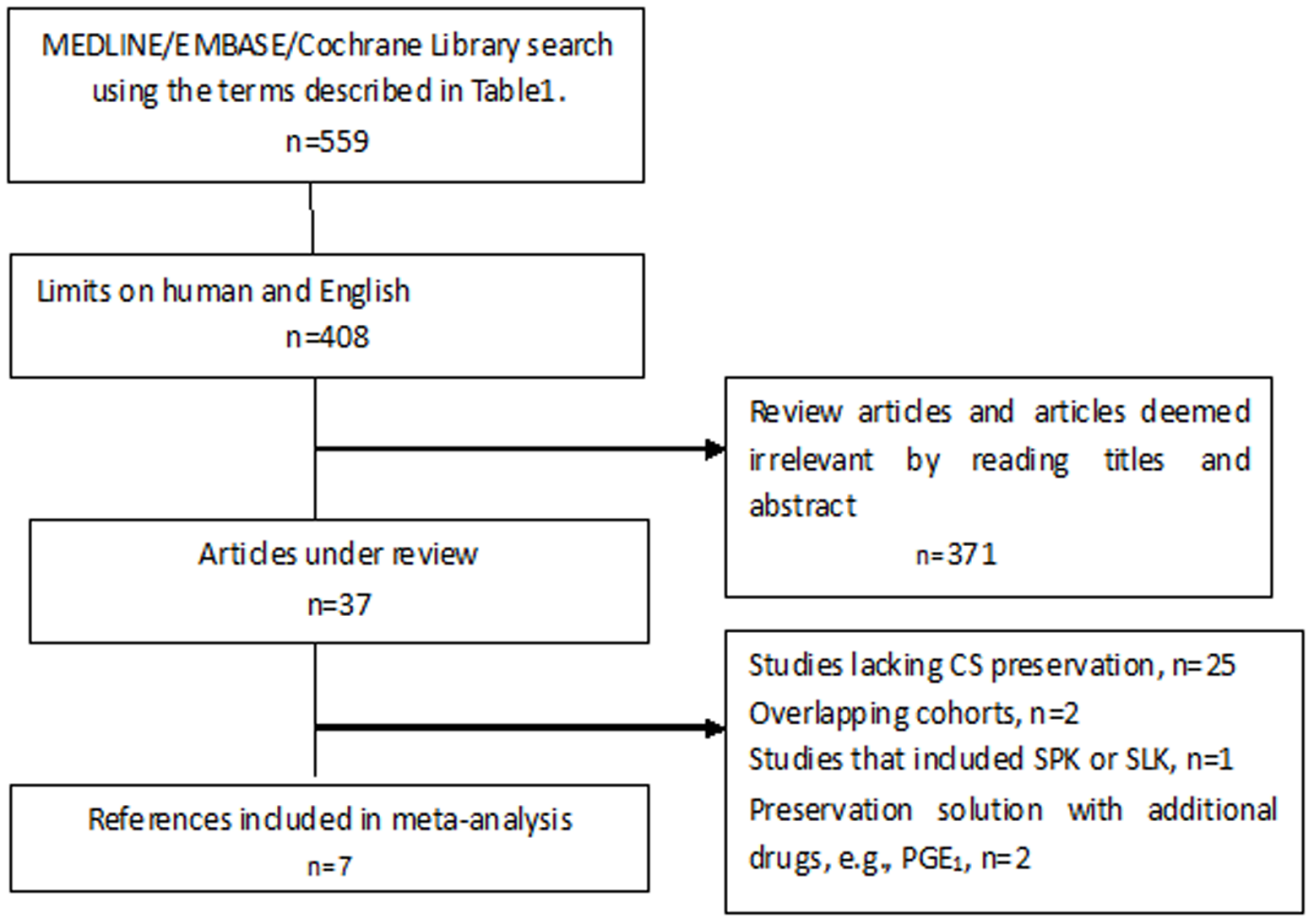
Flow chart illustrating papers selected for analysis.

A search of the PubMed/Medline, Embase and Cochrane library databases was performed. The search strategies are listed in Table 1, and the process of identifying papers for inclusion is shown in Figure 1. The search was conducted in March and April 2013. A manual search of the references of the relevant publications was also performed.

**Table 1:** Search strategies.

Procedure	Contents
1	expanded criteria donors OR ECD
2	machine perfusion OR MP
3	pulsatile machine perfusion OR PMP
4	pulsive perfusion OR PP
5	hypothermic pulsatile perfusion OR HPP
6	pulsatile perfusion preservation OR PPP
7	pulsatile machine perfusion OR PMP
8	cold storage OR static cold storage OR CS
9	1 AND (2–7) AND 8

### Selection criteria

Studies reporting outcomes of ECD kidney transplantation using HMP preservation versus CS were included in this meta-analysis. Exclusion criteria were: (1) overlapping studies from the same institution (avoid duplication);(2) studies that included kidneys from simultaneous kidney-pancreas (SKP)transplants and simultaneous kidney-liver(SLK) transplants; (3) HMP or SC solutions with additional drugs, e.g., PGE_1_;(4) study that contain both ECD and other data, such as donation after cardiac death (DCD), but ECD data cannot be separated apart;and (5) animal studies, review articles, studies in languages other than English.

### Quality assessment

The publications were reviewed and data were extracted by two independent investigators with disagreements resolved through discussion and consensus. The individual studies were evaluated by the Downs and Black quality assessment method[16], which is a list of 27 criteria for evaluating both randomized and nonrandomized comparative studies. Studies could divided into 5 different aspects:reporting, external validity, bias, confounding and power and reach a maximum of 24 points.

### Data synthesis and analysis

Pooled odds ratios (ORs) were used to evaluate the event rates, and the results were reported with 95% confidence intervals (CIs).A P value <0.05 was considered a significant difference between the two groups. Heterogeneity in all of the included studies was evaluated by*Χ^2^* and*Ι^2^* statistical tests. A random effect model was adopted when P<0.05 or*Ι^2^*>50%, and a fixed-effect model was used when P>0.05 or*Ι^2^*<50%. However, taking into account the presence of non-RCTs and different sample size of the included studies, a sensitivity analysis was performed to compare the incidence of DGF and PNF between HMP and CS preservation. A funnel plot is designed to check the existence of publication bias in systematic reviews and meta-analyses. All statistical analyses were performed with Review Manager (RevMan version 5.1, 2008. The Nordic Cochrane Centre, Rigshospitalet).

## Results

### Search results and included studies

Based on the search strategies and selection criteria, we included seven studies comparing HMP to CS in this review [5], [17]–[22], involving 2374 and 8716 kidney grafts with HMP and CS preservation respectively, all from ECD donors. Of these seven studies, two were randomized controlled trials(RCTs), one was a prospective study, and four were retrospective studies. Two of the studies were from Germany, one was from France, one was from Sweden, while the remaining three were from the USA. The years of publication spanned from 2006 to 2013. The study characteristics are shown in Table 2, and the primary outcomes appear in Table 3.

**Table 2:** Characteristics of the included studies.

References	Institution	Study design	Study period	HMP model	Sample size		Solution		CIT (hours)		Recipient age (years)		Initial immunosuppression
									Mean(range)		Mean(range)		
					HMP	CS	HMP	CS	HMP	CS	HMP	CS	
Sedigh, A 2013[22]	Sweden, single-center	Retrospective	6.2010–7.2012	LifePort	36	59	KPS-1	UW	12.8 (7.0–24.5)	11.7 (5.3–25.0)	61 (22–57)	58 (24–79)	Tacrolimus
Gallinat, A(2012)[17]	Germany, multicenter	RCT	NA	LifePort	85	85	KPS-1	UW or HTK	11 (4–22)	10.5 (3–24)	66 (39–79)	66 (37–79)	N/A
Treckmann, J (2011)[18]	Germany multicenter	RCT	11.2005–10.2006	LifePort	91	91	KPS-1	UW or HTK	13 (3–23)	13 (4–29)	65 (20–79)	65 (32–79)	N/A
Abboud, I (2011)[5]	France, single-center	Prospective	2.2002–9.2009	LifePort	22	22	KPS-1	UW	20.3 (12.2–28.4)	22.2 (12–32.4)	56.5±11.0	53.0±15.4	ALG
Stratta, R(2007)[20]	USA, single-center	Prospective	11.2001–11.2006	RM3	114	27	KPS-1	KPS-1	24.5 (16.4–32.6)	19(13.3–24.7)	55.4±11.0	60.1±9.6	104 ALG; 37alemtuzumab
Matsuoka, L (2006)[21]	UNOS	Prospective	01.2001–12.2003	N/A	912	3706	N/A	N/A	20.1 (11.2–29)	18.9(10.8–27)	56±11.4	54.5±12.3	N/A
Buchanan, P. M (2008)[19]	USRDS	Prospective	1995–2004	N/A	1114	4726	N/A	N/A	19.9 (11.5–28.2)	20.9(12.2–29.6)	N/A	N/A	N/A

RCT, randomized controlled trial; HMP, hypothermic machine perfusion; CS, cold storage; CIT, cold ischemic time; HTK, histidine–tryptophan–ketoglutarate;WIT, warm ischemic time; N/A, unavailable; ALG, antilymphocyte globulin; KPS-1, kidney preservation solution-1; UW, University of Wisconsin.

**Table 3:** Main outcomes of the included studies.

References	DGF			PNF			1-yr graft survival			1-yr patient survival		
	HMP(%)	CS(%)	p	HMP(%)	CS(%)	p	HMP(%)	CS(%)	p	HMP(%)	CS(%)	p
Gallinat, A (2012)[17]	29.4	34.1	0.58	3.5	12.9	0.02	89	81	0.139	94.1	95.2	0.79
Treckmann, J(2011)[18]	22	29.7	0.27	3	12	0.04	92.3	80.2	0.02	93.4	96.7	0.3
Abboud, I(2011)[5]	9	31.8	0.021	0	4.5	0.312	95	91	NS	95	95	NS
Matsuoka, L(2006)[21]	25.8	37.1	0.001	2.6	3.2	0.37	N/A	N/A	N/A	N/A	N/A	N/A
Stratta, R(2007)[20]	11	37	0.002	3	4	NS	N/A	N/A	N/A	N/A	N/A	N/A
Buchanan, P. M(2008)[19]	26.9	38	0.0001	N/A	N/A	N/A	N/A	N/A	N/A	N/A	N/A	N/A
Sedigh, A(2013)[22]	16.7	20.3	0.658	N/A	N/A	N/A	N/A	N/A	N/A	N/A	N/A	N/A

HMP, hypothermic machine perfusion; CS, static sold storage; DGF, delayed graft function; PNF, primary non-function; N/A, not-available; NS, not significant.

### Quality of included studies

The results of the evaluation criteria adapted from Downs and Black [16] are shown in Table 4. The scores were, on average, 16.7 points (SD = 3.0). The lowest score was 14 points[22], whereas one study reached the highest score of 22 points[18].

**Table 4:** Evaluation criteria adapted from Downs and Black (1998).

References	Reporting 0–10	External validity 0–2	Bias 0–7	Confounding 0–4	Power 0–1	Overall 0–24
						
Sedigh,A(2013)[22]	6	1	5	2	0	14
Gallinat, A (2012)[17]	8	1	6	5	0	20
Treckmann, J(2011)[18]	9	2	6	5	0	22
Abboud, I(2011)[5]	6	1	5	2	0	14
Stratta, R(2007)[19]	7	2	5	2	0	16
Matsuoka, L(2006)[21]	8	1	5	2	0	16
Buchanan, P. M(2008)[19]	7	1	5	2	0	15
**Mean (SD)**	7.3(1.1)	1.2(0.5)	5.2(0.49)	2.8(1.4)	0(0)	16.7(3.0)

### Outcomes

**Figure 2:**
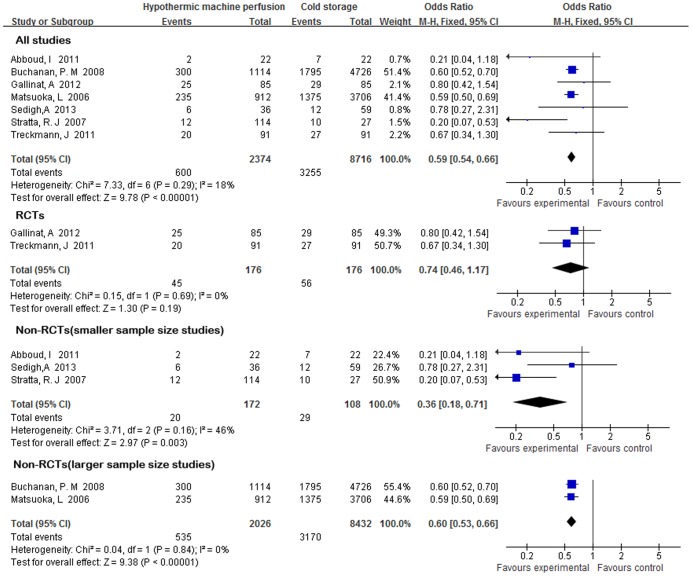
DGF rates for ECD kidneys preserved by HMP versus CS.

All seven studies reported the incidence of DGF. All of the studies defined DGF as the need for dialysis within the first week post-transplant. Heterogeneity was evident but not statistically significant among the studies (*Χ^2^* = 7.33,P = 0.29, *I^2^* = 18%), thus a fixed-effect model was adopted. The incidence of DGF was significantly reduced in the HMP preservation compared with CS (OR = 0.59; 95% CI 0.54–0.66; P<0.001) (Figure 2). Due to heterogeneity in study design and sample size, sensitivity analyses were conducted using the two RCTs and larger sample size studies respectively. Evaluating the two larger size studies [19], [21],the protective effect was still found with an OR of 0.60(95% CI 0.53–0.66), with no heterogeneity(*Χ^2^* = 0.04,P = 0.84, *I^2^* = 0%),fixed-effect model. However, we found that the incidence of DGF was not significantly different between HMP and CS preservation using the two RCTs (OR = 0.74; 95% CI 0.46–1.17; P = 0.19) (Figure 2).

**Figure 3:**
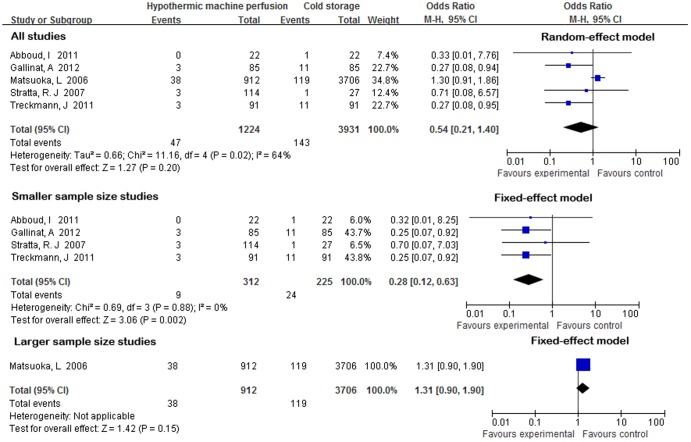
PNF rates for ECD kidneys preserved by HMP versus CS.

**Figure 4:**
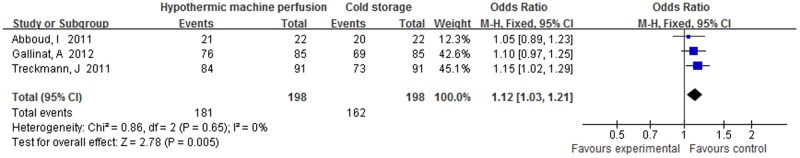
One-year graft survival for ECD kidneys preserved by HMP versus CS.

**Figure 5:**
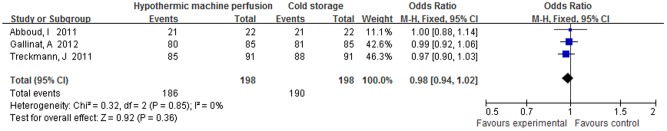
One-year patient survival for ECD kidneys preserved by HMP versus CS.

Five studies reported the incidence of PNF after transplantation[5], [17], [18], [20], [21]. One study defined PNF as a permanent lack of function of the allograft from the time of transplantation[5]. The other four studies did not provide a definition of PNF. Heterogeneity was identified (*X^2^* = 11.45, P = 0.02; *I^2^* = 65%), thus a random-effect model was adopted. The incidence of PNF was not significantly different between HMP and CS preservations (OR = 0.54; 95% CI 0.21–1.40; P = 0.20) (Figure 3). Due to heterogeneity in sample size, a sensitivity analysis was conducted using the smaller size studies. However, we found that the incidence of PNF was significantly lower in HMP preservation compared to CS, with an OR of 0.28(95% CI 0.12–0.63), with no heterogeneity(*Χ^2^* = 0.69,P = 0.88, *I^2^* = 0%),fixed-effect model(Figure 3). The same three studies reported the incidence one-year graft survival[5], [17], [18]. None of the study provided a definition of one-year graft survival. There was no heterogeneity (*X^2^* = 0.86,P = 0.65, *I^2^* = 0%); thus, a fixed-effect model was adopted. There was a trend favoring the use of HMP, the one-year graft survival rate was significantly different between HMP and CS preservation (OR = 1.12; 95% CI 1.03–1.21; P = 0.005) (Figure 4). The same three studies reported the incidence of one-year patient survival[5], [17], [18]. None of the study provided a definition of one-year patient survival. No heterogeneity was identified (*X^2^* = 0.32, P = 0.85, *I^2^* = 0%);thus, a fixed-effect model was applied. However, the incidence of one-year patient survival was not significantly different between HMP and CS preservation (OR = 0.98; 95% CI 0.94–1.02; P = 0.36) (Figure 5).

### Publication bias

We took no formal steps to determine publication bias, such as plotting effect sizes or calculating test statistics, because any formal method would have had little power given the small number of studies.

## Discussion

The use of ECD kidneys in older patients has become a common practice over the last decade, with recipients 50 years of age and older receiving 70% of these kidneys[23]. HMP can reduce warm-ischemia injury, and it provides an interesting opportunity to evaluate kidney graft quality before transplantation[15]. Recently three meta-analyses compared HMP with cold storage [24]–[26], finding that HMP could reduce the DGF rate, but the PNF incidence and one-year graft and patient survival rates were not different in patients using the two preservation methods. However, these articles focused on normal kidney donors, none of them was with regard to ECD kidney transplantation.

This meta-analysis showed that HMP significantly reduced the incidence of DGF, although the sensitivity analysis could not determine a significant difference using only the RCTs. In the multicenter RCT included in this meta-analysis, Treckmann, J et al concluded that HMP significantly reduced the risk of DGF compared with CS (OR 0.460, P = 0.047)[18]. No significant difference could be drawn using the only 2 RCTs because the sample size was small. DGF is an early indicator for organ quality and preservation. In 2009, Cyril Moers et al conducted an RCT using a paired design, in which both kidneys were from the same donor, with one kidney undergoing HMP and the other CS; they showed a significant reduction in the DGF rate of 26.5% in the HMP preservation group compared with 20.8% in CS [27]. In a retrospective single-center analysis of 141 ECD kidneys, Stratta et al reported a remarkable reduction in the rate of DGF with HMP preservation (11%) versus CS(37%)[11]. Schold et al. examined the Scientific Registry of Transplant Recipients (SRTR) database from 1994 to 2003, compared HMP with CS in ECD kidneys transplantation and found that the rates of DGF were 20% with HMP preservation and 28% in CS. The study also examined paired transplanted kidneys, finding that HMP preservation significantly decreased the DGF rate compared with CS (19% vs. 26%, p<0.001) [28]. The incidence of DGF in ECD kidneys differed in each study; one important reason for this might be the length of cold ischemic times in each study [21], [27]. Cold ischemia time was a risk factor for DGF in ECD kidney transplants [29].

DGF was shown to be a risk factor for graft failure after kidney transplantation[30].The sensitivity analysis found that HMP had a protection effect in reducing the PNF rate using the smaller sample size studies. However, Matsuoka, L et al [21] retrospectively analyzed the data from United Network for Organ Sharing (UNOS), which contained 4618 ECD kidneys, and found that HMP could not decrease PNF rate compared to CS (2.6% versus 3.2%, p = 0.32).Taking the larger sample size study into account, we found that HMP preservation could not improve primary non-function(PNF)for recipients receiving HMP kidneys. Unlike other meta-analysis[24]–[26],we found that HMP preservation could improve one-year graft survival rate compared to CS preservation. Polyak et al. found that one-year graft survival was greater with ECD kidneys that were preserved by HMP compared with CS (88% vs. 79%, p = 0.02)[31]. Treckmann, J et al. conducted an international randomized controlled study and obtained similar conclusion (92.3% vs. 80.2%, P = 0.02) [18].However, we found that HMP preservation was not associated with improvement of one-year patient survival. ECD kidneys often associated with deteriorated function and more frequent DGF, but these factors did not increase the mortality of the recipients[32].

A number of factors might have confounded the interpretation of this meta-analysis. First, there was heterogeneity between study design, sample size and the years covered. This meta-analysis contained only two RCTs on this special subject. Although the nonrandomized studies were subject to lower quality, which might have resulted in an unbalanced selection of patients, they provided the best evidence available on this subject. Second, the pump parameters, such as perfusion pressure, type of perfusate used and cold storage solution, varied and were not always clearly reported(Table 2). Third, we observed that the recipient populations, the length of cold ischemic time and the use of immunosuppressive agents were variable and that the heterogeneity observed in clinical trials was correlated with the habits and preferences of individual institutions(Table 2).

Usage of HMP could reduce the discard rate of ECD kidneys from 40% to 30%and decrease DGF risk for ECD kidneys with longer cold ischemia time(>30 hours), minimizing postoperative complications and maximizing organ utilization[28]. HMP also provides a quantitative assessment of renal vasospasm due to the pump parameters that are generated during machine preservation [20]. In addition, rather than increasing direct costs to the transplant program, HMP was correlated with lower costs for transplant hospitalizations, likely due to the associated reduction in DGF[19]. Gómez, C conducted a cost-effectiveness assessment for ECD kidney transplantation and found that the introduction of HMP cost $505, however, $3,369 was savedin each DGF or PNF case[33].

The meta-analysis demonstrates that HMP is associated with a reduced incidence of DGF and increased one-year graft survival rate compared to CS for ECD kidney transplantations, but it was not associated with the incidence of PNF and one-year patient survival.

## Supporting Information

Checklist S1 (DOC)

Prisma 2009 Flow Diagram S1 (DOC)
