# Supplementary material for: Hypothermic Machine Perfusion Reduces Delayed Graft Function and Improves One-Year Graft Survival of Kidneys from Expanded Criteria Donors: A Meta-Analysis
Source: PLoS One. 2013 Dec 10;8(12):e81826. doi: 10.1371/journal.pone.0081826 (PMC3858268; doi:10.1371/journal.pone.0081826)
Supplement: Prisma 2009 Flow Diagram S1 — (DOC) [file pone.0081826.s002.doc]

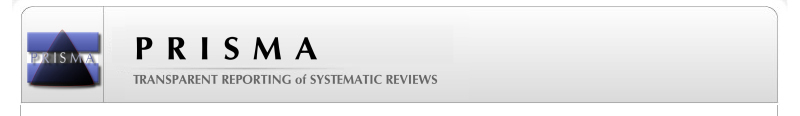
**PRISMA 2009 Flow Diagram**

**Screening**

**Included**

**Eligibility**

**Identification**

Records identified through database searching
(n =559 )

Additional records identified through other sources
(n = 0 )

Records after duplicates removed
(n = 559 )

Records screened
(n = 408 )

Records excluded
(n = 371 )

Full-text articles assessed for eligibility
(n =37 )

Full-text articles excluded, with reasons
(n = 30 )

Studies included in qualitative synthesis
(n = 7 )

Studies included in quantitative synthesis (meta-analysis)
(n = 7 )
